# Optimizing TLIF Approach Selection: An Algorithmic Framework with Illustrative Cases

**DOI:** 10.3390/jcm14124209

**Published:** 2025-06-13

**Authors:** Alyssa M. Bartlett, Summer Shabana, Caroline C. Folz, Mounica Paturu, Christoper I. Shaffrey, Parastou Quist, Olumide Danisa, Khoi D. Than, Peter Passias, Muhammad M. Abd-El-Barr

**Affiliations:** 1 Department of Neurosurgery, Duke University Medical Center, Durham, NC 27710, USAkhoi.than@duke.edu (K.D.T.);; 2 Division of Spine Surgery, Department of Orthopaedic Surgery, Duke University Medical Center, Durham, NC 27710, USA; olumide.danisa@duke.edu

**Keywords:** transforaminal lumbar interbody fusion, surgical decision-making, Kambin’s triangle, spine segmentation

## Abstract

Transforaminal lumbar interbody fusion (TLIF) is a commonly employed surgical technique for managing lumbar degenerative disease and spinal instability. While it offers advantages over posterior lumbar interbody fusion (PLIF), traditional TLIF often involves prolonged recovery and morbidity due to muscle retraction. To improve outcomes, several alternative techniques have emerged, including minimally invasive TLIF (MIS-TLIF), trans-Kambin percutaneous TLIF (PE-TLIF), and transfacet TLIF (TF-TLIF). Each approach presents distinct anatomical and technical advantages, yet no standardized framework exists to guide their selection based on individual patient anatomy. In this study, we review the evolution of TLIF techniques and propose a novel algorithm that integrates patient-specific imaging, anatomical variability, and segmentation data to guide surgical decision-making. By analyzing the surgical corridors, indications, and limitations of each approach, and presenting representative clinical cases, we demonstrate how this algorithm can be applied in practice. For instance, TF-TLIF may be optimal in patients requiring direct decompression without major deformity, while PE-TLIF may be appropriate for those with Kambin’s triangles measuring ≥ 9 mm, allowing for indirect decompression. This tailored framework aims to optimize outcomes and reduce complications. Further prospective validation and incorporation of AI-driven segmentation tools are needed to support broader clinical implementation.

## 1. Introduction

The transforaminal lumbar interbody fusion (TLIF) emerged in 1982 as a safer alternative to the posterior lumbar interbody fusion (PLIF) for the treatment of lumbar degenerative disease and spinal instabilities. By utilizing a transforaminal approach for circumferential fusion, TLIF reduces neural retraction, lowers the risk of iatrogenic injury, and preserves contralateral structures for fusion while minimizing associated morbidities [1]. However, despite being an improvement over PLIF, the prolonged muscle retraction required for traditional open TLIF is associated with extended recovery periods and long-term paraspinal muscle degeneration [2].

To overcome these challenges, several TLIF variations have emerged, including minimally invasive TLIF (MIS-TLIF), trans-Kambin’s percutaneous endoscopic TLIF (PE-TLIF), and transfacet TLIF (TF-TLIF). MIS-TLIF employs paramedian incisions, tubular retractors, and image guidance to minimize soft tissue disruption [3]. This less invasive technique is particularly beneficial in cases of facet hypertrophy, central stenosis, disc herniation, and facet cysts, though it comes at the expense of reduced direct visualization and increased radiation exposure [4,5,6]. PE-TLIF accesses the disc space percutaneously via Kambin’s triangle using endoscopic guidance for discectomy and cage placement [7]. This technique alleviates nerve root compression without direct decompression and avoids extensive bone resection, making it particularly effective for treating spondylolisthesis and degenerative disc disease. In recent years, refinements in endoscopic technology have further improved visualization, ergonomics, and patient outcomes in PE-TLIF, broadening its indications and safety profile. Finally, TF-TLIF involves resecting the facet joint, allowing direct access to the neural foramen and disc space while minimizing neural retraction [8]. As a recent modification to the MIS-TLIF, the TF-TLIF lacks long-term clinical outcome data, though early reports are promising [9].

Ultimately, surgeon preference and experience primarily determine the choice of approach, as no standardized criteria exist to tailor techniques to individual patient anatomy and pathology. A practical framework to guide spine surgeons in selecting the most appropriate TLIF approach is needed. We introduce a decision-making algorithm guided by symptomatology and anatomical feasibility assessments. This paper explores TLIF techniques, their integration, and the incorporation of innovative technologies such as endoscopy and patient-specific segmentation in a structured decision-making framework.

## 2. Surgical Approaches

### 2.1. MIS-TLIF

MIS-TLIF is performed through a small paramedian incision using tubular retractors and fluoroscopic guidance. After muscle dilation, a laminectomy and partial facetectomy are performed to access the disc space via a transforaminal route. The disc material is removed, and the endplates are prepared. An interbody cage packed with autograft or allograft is inserted into the disc space to restore disc height and promote fusion. Percutaneous pedicle screws are then placed bilaterally, and rods are secured to provide segmental stability. The technique minimizes muscle disruption and postoperative pain while achieving solid fusion, but may require longer operations and higher radiation exposure [10]. Multiple studies have demonstrated that MIS-TLIF achieves comparable fusion rates and clinical outcomes to open TLIF, with the added benefits of reduced blood loss, shorter hospital stays, and lower postoperative pain, though it may have a higher learning curve and increased radiation exposure [11,12]. Recent integration of intraoperative navigation, robotic assistance, and augmented reality tools has enhanced the precision of MIS-TLIF, improving screw placement accuracy and reducing both operative time and radiation exposure [13]. Additionally, MIS-TLIF has demonstrated utility in revision surgeries and multilevel disease, where prior scar tissue or altered anatomy complicates open re-entry. While technically demanding, careful planning and navigation allow for safe access and instrumentation, expanding the role of MIS approaches beyond single-level pathology [4,6]. Despite its advantages, MIS-TLIF may be less suitable for patients with severe central canal stenosis, complex anatomical distortions, or morbid obesity, as these factors can limit visualization and necessitate prolonged fluoroscopic exposure, increasing operative complexity and radiation burden [11,12,14].

### 2.2. TF-TLIF

The TF-TLIF approach utilizes a posterior paramedian incision and limited bony resection to remove the facet joint unilaterally and access the disc space without a wide laminotomy or medial retraction of the thecal sac. Compared to MIS-TLIF, which starts medial for the laminotomy before proceeding laterally for decompression, TF-TLIF begins with facetectomy. After facetectomy, the disc is evacuated through the transfacet corridor, and the endplates are prepared. Importantly, the traversing and/or exiting nerve roots are not exposed. A cage with autograft or allograft is placed in the intervertebral space, and pedicle screw instrumentation is inserted through the same or adjacent incisions. If a direct decompression is needed, it can be performed after the cage placement. This approach preserves more of the posterior midline structures and can reduce operative time and blood loss compared to standard TLIF [8,15]. The use of expandable interbody cages in TF-TLIF has been shown to optimize restoration of disc height and segmental lordosis while minimizing neural retraction, allowing for safer and more effective instrumentation through a narrow corridor [16,17]. This approach may be particularly beneficial for patients with elevated surgical risk, such as those with coagulopathies like von Willebrand disease or obesity, where minimizing soft tissue dissection and blood loss is paramount [15,18]. However, TF-TLIF may be technically challenging in patients with high-grade spondylolisthesis, severe coronal or sagittal deformity, or calcified posterior elements, where visualization and access are more limited despite the minimally invasive approach [3].

### 2.3. PE-TLIF

This technique is performed using an endoscope through a small posterolateral incision, often under local or epidural anesthesia with conscious sedation. After sequential dilation, a working channel is placed, and the endoscope provides visualization of the facet and disc space. A foraminoplasty and partial facetectomy are performed endoscopically to create a safe corridor to the disc. The disc material is removed, endplates are prepared, and an expandable interbody cage with autograft or allograft is inserted. Percutaneous pedicle screws are then placed using image guidance. This ultra-minimally invasive method reduces tissue disruption, blood loss, and recovery time, but has a steeper learning curve, which may increase complication rates compared to other MIS approaches [19]. As PE-TLIF can be performed without induction of general anesthesia, this approach may be especially beneficial for elderly or medically frail patients who are at higher risk for complications from general anesthesia [19]. Compared to MIS-TLIF and TF-TLIF, PE-TLIF typically results in lower intraoperative blood loss, shorter hospital stays, and quicker return to baseline function, but is associated with longer operative times and higher intraoperative radiation exposure due to reliance on fluoroscopy and endoscopy [19]. Despite its technical challenges, PE-TLIF has demonstrated comparable fusion rates to traditional and MIS-TLIF techniques, with systematic reviews confirming its efficacy in reducing back and leg pain while achieving high rates of radiographic fusion [19,20]. While PE-TLIF offers significant advantages in minimizing tissue trauma, the steep learning curve, limited tactile feedback, and risk of exiting nerve root injury may limit its widespread adoption and necessitate careful patient selection [21,22].

While each TLIF variant has demonstrated efficacy in treating lumbar degenerative disease, they differ significantly in terms of technical complexity, visualization, tissue disruption, and recovery profiles. MIS-TLIF strikes a balance between direct decompression and reduced morbidity, while TF-TLIF offers a streamlined corridor for interbody placement with minimal neural manipulation. In contrast, PE-TLIF provides the least invasive access route, ideal for select patients with favorable anatomy and low comorbidity burden. Ultimately, successful outcomes depend on patient selection, anatomical considerations, and surgeon familiarity with each technique, reinforcing the need for an individualized, algorithm-driven approach to TLIF selection.

## 3. Algorithm for TLIF Approach Selection

**Figure 1:**
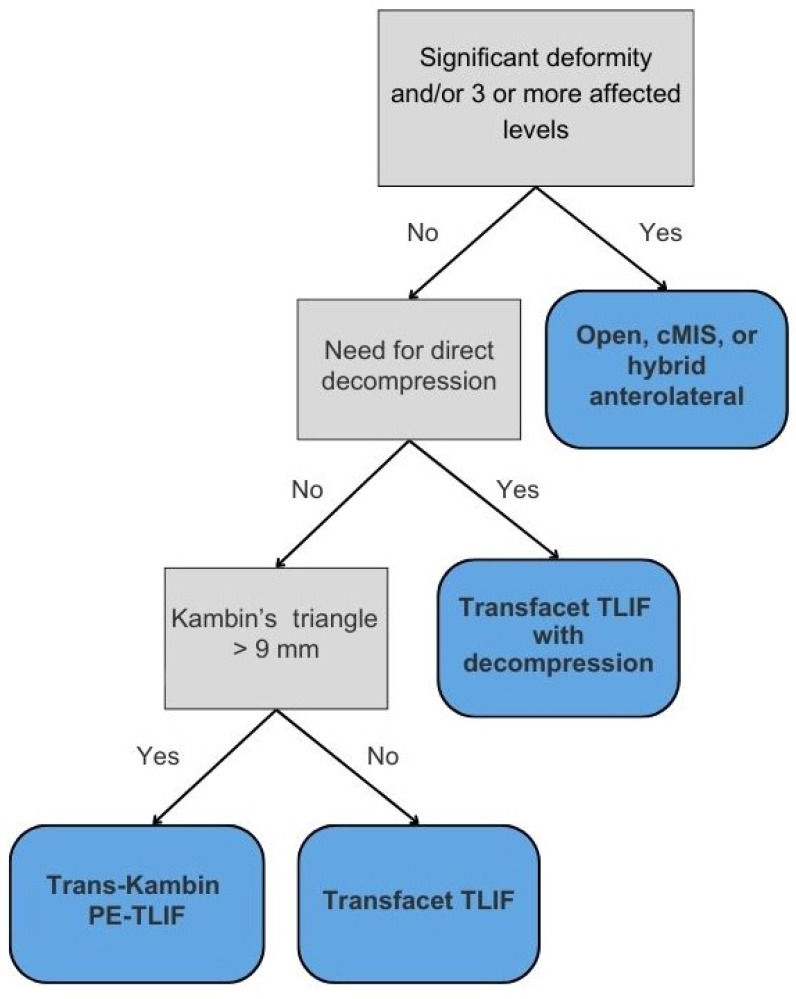
Algorithm for TLIF selection.

Given the evolution and continued optimization of the TLIF, multiple lumbar fusion approaches may be feasible solutions for patients with spondylolisthesis, spine deformity, and other spine pathologies. However, there are no defined guidelines for making these decisions, leaving each proposed surgical approach up to the individual surgeon. This algorithm, based on our institutional experience, creates a framework for surgical decision-making dependent on spine pathology and advanced imaging in the hopes of deciding on the approach to lumbar interbody fusion for each patient (Figure 1).

### 3.1. The Role of Significant Deformity

While TLIF remains a widely used technique for single- or dual-level pathology due to its posterior-only access and ability to restore disc height and spinal stability, its limitations become apparent in cases of complex deformity correction, sagittal imbalance, or extensive degenerative disease. In cases with PI-LL mismatch greater than 30 degrees and/or pre-existing multilevel instrumentation, open approaches provide superior correction of spinopelvic parameters and direct visualization, though at higher morbidity due to incorporating osteotomies and extensive instrumentation [23,24,25].

The circumferential MIS (cMIS) approach yields similar improvement as open approaches, with long-term positive clinical outcomes and less blood loss [26,27]. Beneficial in degenerative scoliosis and flatback syndrome, cMIS may be a promising choice for patients at a higher risk for complications in open surgery. Hybrid anterolateral approaches to deformity correction or multilevel spine pathology have also demonstrated superior deformity correction relative to posterior-only approaches [28]. While posterior-only methods may achieve similar results, an additional osteotomy is required, which carries an increased risk of durotomy and neurologic deficits [29]. Given these considerations, our framework maintains open or hybrid approaches as the default recommendation in cases of complex deformity or sagittal imbalance, recognizing their unparalleled capacity for global alignment correction despite increased surgical burden.

### 3.2. Decompression

The first decision point in our algorithm is whether direct decompression is needed. Direct decompression through laminectomy, discectomy, or foraminotomy is typically indicated for patients with severe stenosis, progressive neurologic deficits, or significant radiculopathy. This approach involves physically removing compressive elements, such as herniated discs, osteophytes, or hypertrophic ligaments, to provide immediate neural decompression. In contrast, indirect decompression relieves neural compression by restoring spinal alignment and disc height without directly removing compressive structures. Often utilized in anterior lumbar interbody fusion (ALIF) or lateral interbody fusion (LLIF), this technique may be suitable for patients with mild to moderate stenosis, though patients with less disc height loss may do well even in cases of severe central canal stenosis [30]. Furthermore, LLIF may confer an advantage of lower cage subsidence rates compared to TLIF in patients with osteoporosis, owing to the larger surface area of LLIF cages [31]. Thus, in osteoporotic patients needing indirect decompression, lateral approaches should be strongly considered.

The decision of whether to perform direct versus indirect decompression depends on multiple factors, including patient symptomatology, the severity and location of neural compression, spinal stability, patient comorbidities, and surgeon preference. While indirect decompression offers a less invasive approach with potentially quicker recovery times, it may be inadequate for patients with severe spondylolisthesis or calcified compressive pathology. In contrast, direct decompression provides immediate relief of radiculopathy but carries a higher risk of nerve root injury. Overall, direct decompression is preferred when there are compressive pathologies such as osteophytes, cysts, or herniated discs causing a radiculopathy [32]. There is also evidence that direct decompression may be the better option in bony lateral recess stenosis [33].

**Figure 2:**
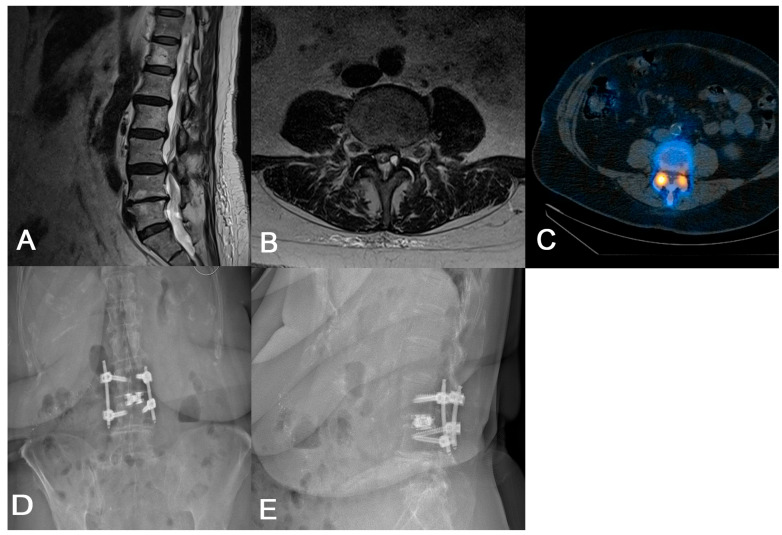
(**A**) Preoperative sagittal lumbar MRI demonstrating L3/4 spondylolisthesis. (**B**) Preoperative axial lumbar MRI showing a cyst on the L3/4 facet in addition to nerve root compression. (**C**) Preoperative axial CT SPECT showing increased radiotracer uptake in the bilateral L3/4 facet joints. (**D**,**E**) Postoperative AP and lateral lumbar radiographs demonstrating appropriate implant placement.

If the surgeon feels that direct decompression is needed, we advocate for a TF-TLIF followed by direct decompression. The TF-TLIF approach provides a novel approach to interbody fusion by accessing the intervertebral disc space entirely through the facet joint, minimizing the risk of iatrogenic nerve injury due to protection of the inferior articular process while still offering long-term improvements in patient disability [8,15]. In one case, a 79-year-old female with a history of von Willebrand disease and chronic refractory low back and bilateral lower extremity radiculopathy was unable to perform activities of daily living without assistance due to her pain (Figure 2). Imaging revealed multilevel degenerative disease with spondylolisthesis at L3/4 as well as a left-sided synovial cyst at the facet. Later CT SPECT of the lumbar spine found increased radiotracer uptake at the right L3/4 facet. Given her radiculopathy and synovial cyst, the patient was agreeable to L3/4 TF-TLIF with direct decompression, as it requires less bony resection compared to MIS-TLIF and protects the exiting and traversing nerve roots. During this case, pedicle screw placement, discectomy, and interbody implant insertion occurred prior to laminectomy for direct decompression. While the patient initially reported some paresthesias in the perioperative period, her pain and radiculopathy improved immediately, with resolution of her paresthesias within 6 months. Similarly, two case series totaling 88 patients undergoing TF-TLIF reported significant long-term improvements in patient-reported pain and functional status [15,18]. While direct decompression was needed in this case due to her radiculopathy, if it is felt that direct decompression is unnecessary, we advocate for the use of advanced segmentation to understand if a trans-Kambin or transfacet approach should be undertaken. Ultimately, careful preoperative planning is essential to determine the optimal surgical approach for each patient.

### 3.3. Measuring Kambin’s Triangle

**Figure 3:**
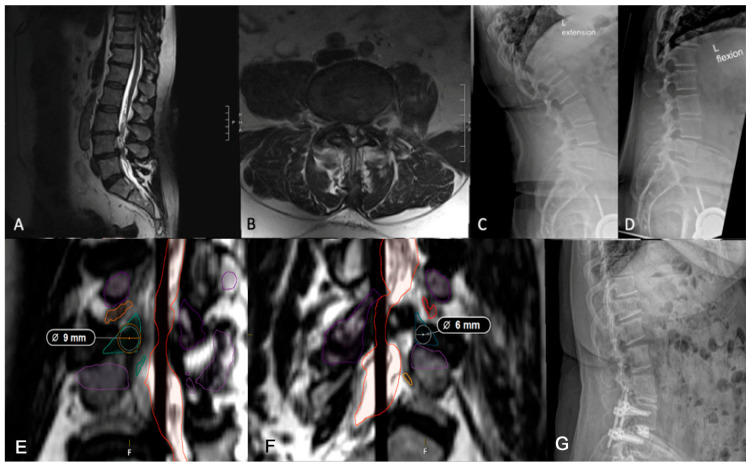
(**A**,**B**) Preoperative lumbar MRI demonstrating L4/5 spondylolisthesis. It should be noted that the patient did not complain of a radiculopathy, despite a small-sided left facet cyst at L4/5. (**C**,**D**) Preoperative flexion and extension radiographs assessing dynamic instability showing grade I spondylolisthesis at L4/5. (**E**) Axial imaging showing a right-sided working corridor measuring 9 mm. (**F**) Left-sided corridor measuring 6 mm, supporting a right-sided approach. (**G**) Postoperative lumbar radiograph demonstrating appropriate implant placement.

**Figure 4:**
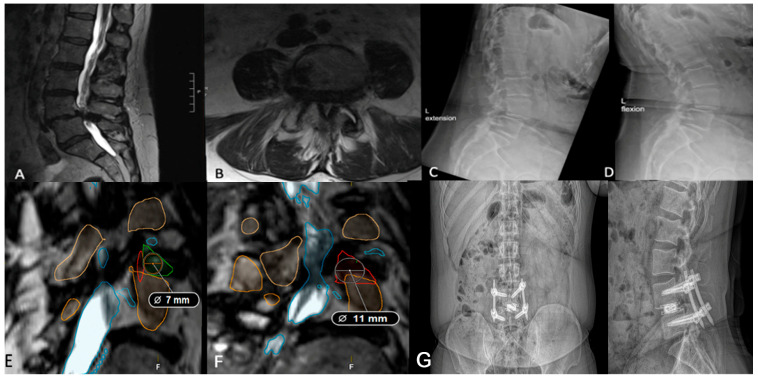
(**A**,**B**) Preoperative lumbar MRI of a patient with grade II L4/5 spondylolisthesis and severe low back pain refractory to conservative management. (**C**,**D**) Preoperative flexion and extension radiographs assessing segmental instability. (**E**) Axial segmentation demonstrating a 7 mm trans-Kambin working corridor. (**F**) Ipsilateral corridor measuring 11 mm, suggesting the TF-TLIF may be appropriate. (**G**) Postoperative standing lateral and anteroposterior radiographs demonstrating appropriate implant placement following TF-TLIF.

Kambin’s triangle—bordered by the exiting nerve root, proximal endplate, and articular process of the lower vertebra—and the traversing nerve root and dural sac, is devoid of critical neurovascular elements, allowing safe access to the spine [34,35]. Preoperative evaluation of Kambin’s triangle can be performed using advanced imaging and segmentation software for three-dimensional reconstruction of the lumbar spine [36]. Using axial and sagittal T2-weighted MRI scans acquired at 1 mm slice thickness, the triangle can be delineated by identifying the borders. Then, segmentation tools, such as 3D slicer (Brigham and Women’s Hospital, Boston, MA, USA) or Smartbrush (BrainLab, Munich, Germany), enable manual labeling of the nerve roots, vertebrae, and thecal sac for subsequent measurement of the working corridor. The corridor is measured by outlining the corridor, approximating the largest triangle inside, and calculating the area. In patients whose Kambin’s triangle measures greater than 9 mm, a trans-Kambin approach is reasonable, as this is a common choice of cannula size. Additionally, Tabarestani et al. reported that the mean permissible cannula diameter at lumbar disc spaces below L2 is at least 8.98 mm, suggesting sufficient space to accommodate this approach [37]. Importantly, it may be that one side is larger than the other, and it is advisable to use the larger side. The assessment of operative laterality is a particularly critical factor in optimizing surgical decision-making. Degenerative changes, disc herniation location, and foraminal stenosis can lead to asymmetry in Kambin’s triangle dimensions, which can then be determined preoperatively using segmentation technology [38]. In one case, a preoperative segmentation of L4/5 showed a reduced left-sided working corridor (6 mm) compared to the right (9 mm) (Figure 3). Given the differences in the working corridor, a right-sided PE-TLIF was selected. However, in patients whose Kambin’s triangle does not measure sufficiently to handle a minimum cannula of 9 mm, the TF-TLIF may be an appropriate alternative. Early reports have demonstrated that the TF corridor increases with spine pathology, specifically spondylolisthesis, due to the increasingly oblique trajectory of the exiting nerve root [37]. In another case, preoperative segmentation of a patient with grade II spondylolisthesis without radiculopathy revealed a larger transfacet corridor (11 mm) compared to Kambin’s triangle (8 mm), supporting TF-TLIF without direct decompression as the optimal surgical approach (Figure 4) [39]. This allows for a larger safe working corridor and, subsequently, a larger cannula for instrumentation without additional nerve root retraction. At 6-month follow-up, the patient’s Visual Analogue Score (VAS) for back pain improved from 7 to 0, and his Oswestry Disability Index (ODI) decreased from 14 to 0, aligning with previous findings [15]. As degeneration can create asymmetry, surgeons should aim to preoperatively assess both sides to optimize laterality choice.

## 4. Discussion

TLIF was developed to address the risks associated with PLIF, particularly the need for extensive neural and paraspinal muscle retraction, which increases the risk of durotomy, nerve injury, and failed back surgery syndrome [40,41]. By providing unilateral access to the intervertebral foraminal space, TLIF minimizes direct dissection, reduces surgical trauma, and lowers the risk of iatrogenic injury while preserving biomechanical stability [42,43]. However, traditional open TLIF still poses challenges, including paraspinal muscle injury from prolonged retraction and difficulty in correcting coronal imbalance and restoring lordosis compared to PLIF [42,43,44].

As previously discussed, MIS-TLIF minimizes soft tissue disruption and is suitable for high-risk surgical candidates, such as elderly patients or those with comorbidities associated with increased infection risk, like diabetes or obesity [11,12,14]. Its smaller surgical footprint is associated with shorter hospital stays, less postoperative pain, and reduced blood loss [4,45,46]. The TF-TLIF provides a direct path to the disc space via the facet joint, allowing medialization for formal decompression when needed, and then, if needed, the tube can be medialized more to perform a formal decompression. Trans-Kambin TLIF emerged as a technique that utilizes a safe anatomical working corridor to access the intervertebral disc space. The percutaneous approach of the trans-Kambin PE-TLIF lends the advantage being ultra-minimally invasive.

However, MIS approaches still carry disadvantages, primarily that increased reliance on fluoroscopy results in greater radiation exposure, while limited visualization contributes to complications such as durotomy [36,37]. Although infection risk is lower than open approaches, revision and multilevel MIS cases still carry an elevated risk [46]. Finally, given the limited visualization and potential irritation from intraoperative technologies like cannulas, MIS approaches carry a greater risk of exiting and traversing nerve root injury [21]. The development of new technologies to mitigate these risks has paved the way for patient-specific TLIF using anatomic visualization of surgical corridors to extensively plan trajectories and approaches prior to surgery to reduce the use of intraoperative fluoroscopy and potential iatrogenic injury. In particular, preoperative anatomical planning may be of highest utility in patients undergoing complex or multilevel procedures, where the margin for error is narrower and the consequences of nerve injury or suboptimal cage placement are more severe.

To our knowledge, the proposed framework (Figure 1) is the first structured algorithm specifically designed to guide TLIF approach selection by building upon existing literature while integrating anatomical and clinical considerations. Unlike current approaches that rely heavily on surgeon preference, general MIS vs. open paradigms, or broad clinical indicators, this model introduces objective thresholds to determine candidacy for modifications of the TLIF. For example, while the model retains the standard of performing direct decompression in cases of compressive pathology such as radiculopathy, cysts, or osteophytes, its flexibility in recommending TF-TLIF with or without decompression reflects a growing preference for a ‘cage-first’ approach using expandable cages, which can provide indirect decompression and allow for additional direct decompression in specific cases [8]. While MIS approaches traditionally involve decompression before interbody cage insertion, emerging evidence favors a reverse sequence: inserting the cage first, followed by decompression. Recent studies suggest this sequence may lead to improved outcomes with expandable cages, as these cages lead to better restoration of disc height and segmental lordosis, and less decompression is needed [16,17]. This order reduces the need for bilateral decompression and nerve manipulation, resulting in shorter operative times and decreased nerve root injury risk [16,47]. As such, the TF-TLIF now plays a central role in our surgical algorithm, given ease of access to the disc space. By prioritizing an anatomically informed approach, this framework not only guides access decisions but also aims to reduce complication rates and enhance postoperative recovery.

Importantly, these distinctions are clinically meaningful. Prospective and meta-analysis data suggest that approaches associated with less muscle disruption and minimal nerve manipulation, such as PE-TLIF and TF-TLIF, can lead to faster ambulation after surgery, reduced postoperative pain, and lower 30-day complication rates when appropriately selected [15,48,49]. However, these benefits depend on appropriate patient selection, underscoring the need for anatomically tailored surgical planning.

Furthermore, this model introduces objective anatomical thresholds, such as having a Kambin’s triangle with a diameter greater than 9 mm. This threshold aligns with previous studies highlighting the variability in Kambin’s triangle dimensions and its association with increased risk of exiting nerve root injury in narrower spaces [35,50]. Kambin’s triangle increases in size from L1 to S1, with the largest area found at the L5–S1 level (>100 mm^2^) [35,38]. However, its dimensions vary significantly among patients [34,35,50]. A cadaveric study by Ozer et al. found that only 20% of individuals have the classically wide triangle, suggesting a limited subset of patients have this ideal corridor [50]. In narrower Kambin’s triangles, the risk of exiting and traversing nerve root injury rises due to space confinements, an observation supported by exiting nerve injury being cited as the most common complication of PE-TLIF [22,51]. Despite these risks, studies have shown that PE-TLIF is associated with reductions in back and leg pain and high fusion rates [19,20]. A systematic review similarly found interpatient variation in both triangle size and critical anatomical structures [35]. Furthermore, the size of Kambin’s triangle increases caudally down the vertebral column, especially in diseased segments [35].

To address anatomical variability, efforts are underway to improve preoperative planning. While MIS approaches rely on intraoperative fluoroscopy, small studies have examined the feasibility of using segmentation software to design patient-specific TLIFs appropriate for their anatomy [38]. Currently, surgeons with the available advanced imaging and software may manually segment individual nerve roots from preoperative MRI, mapping the vertebral bodies, nerve roots, and surrounding musculature to determine the size of Kambin’s triangle bilaterally, identify anatomical aberrances, and determine the feasibility of using the PE-TLIF approach. However, widespread implementation of segmentation is limited by variable access to advanced imaging and segmentation software, as well as the initial and ongoing costs associated with these tools. Additionally, there is a learning curve for adopting new software, and integration into existing surgical workflows can be challenging. These barriers may disproportionately affect lower-resource or community-based practices, underscoring the need for scalable, user-friendly options to ensure equitable adoption. While patient-specific TLIFs can be planned by manual nerve segmentation, automated spine segmentation is a priority for the future. Deep learning models have shown promise in facilitating automation, though challenges remain due to anatomical complexity, vertebral shape diversity, and surrounding musculature and subcutaneous fat [52,53]. Still, advancements in imaging and automation are steadily bringing personalized, precision-guided spinal surgery closer to routine practice.

Enhanced planning enables surgeons to select the most feasible approach and proactively adapt to unexpected variations in anatomy, reducing the likelihood of approach-related complications. Moreover, when integrated with neuromonitoring and advanced navigation, such planning can facilitate safer and more accurate screw and cage placement. Ultimately, the convergence of patient-specific modeling, real-time imaging, and improved surgical decision-making frameworks offers a promising avenue to make TLIF safer, more predictable, and more efficient across a range of patient populations.

### Limitations

This paper proposes a novel algorithm for TLIF approach selection based on symptomatology and anatomical feasibility, incorporating recent innovations in surgical technique and preoperative imaging. However, several limitations must be acknowledged. First, this work does not represent a formal clinical study, but rather a descriptive and conceptual framework derived from institutional experience and literature review. As such, the proposed algorithm has not yet been validated through prospective studies, expert consensus, or matched cohort comparisons. Its generalizability and clinical utility will need to be assessed in future investigations, ideally through multicenter trials or Delphi consensus processes. Second, while the algorithm considers anatomical constraints and pathology, it inherently relies on high-quality imaging and advanced imaging modalities such as MRI segmentation, which may not be available in all practice settings. The need for patient-specific planning and advanced imaging tools may limit the feasibility of widespread adoption, particularly in resource-limited environments. Finally, this algorithm does not yet account for all patient-specific variables such as bone density, prior surgery, psychosocial factors, or spinopelvic parameters that may impact surgical decision-making. In addition, the decision to perform staged surgeries, use expandable cages, or integrate other advanced technologies such as navigation or robotics was outside of the scope of this framework but may influence surgical planning. Future work may refine this algorithm through the incorporation of additional objective metrics to support more standardized surgical planning.

## 5. Conclusions

The evolution of TLIF has significantly expanded the surgical armamentarium for treating degenerative lumbar disease, offering multiple approaches tailored to specific patient anatomy and pathology. While traditional TLIF provides substantial advantages over PLIF, its associated risks, including prolonged muscle retraction and limited deformity correction, have led to the development of MIS-TLIF, trans-Kambin PE-TLIF, and TF-TLIF. However, the decision-making process for selecting the most appropriate TLIF approach remains largely dependent on surgeon preference rather than standardized guidelines. Our algorithm addresses the current gap in standardization by providing an anatomy- and symptom-based framework, supported by emerging segmentation and imaging technologies. Future research should focus on validating this algorithm through prospective studies and exploring the role of artificial intelligence in automating preoperative planning. As TLIF techniques continue to evolve, refining selection criteria and expanding data-driven surgical decision-making will be critical to optimizing approach and improving surgical outcomes.

## Data Availability

No new data were created or analyzed in this study. Data sharing is not applicable to this article.

## Abbreviations

The following abbreviations are used in this manuscript:

TLIF	transforaminal lumbar interbody fusion
MIS-TLIF	minimally invasive transforaminal lumbar interbody fusion
PE-TLIF	percutaneous endoscopic transforaminal lumbar interbody fusion
TF-TLIF	transfacet transforaminal lumbar interbody fusion
cMIS	circumferential minimally invasive surgery
LLIF	lateral lumbar interbody fusion
ALIF	anterior lumbar interbody fusion
VAS	Visual Analogue Scale
ODI	Oswestry Disability Index
